# Admission rates in a general practitioner-based versus a hospital specialist based, hospital-at-home model: ACCESS, an open-labelled randomised clinical trial of effectiveness

**DOI:** 10.1186/s13049-018-0492-3

**Published:** 2018-04-05

**Authors:** Christian Backer Mogensen, Ejnar Skytte Ankersen, Mats J. Lindberg, Stig L. Hansen, Jørgen Solgaard, Pia Therkildsen, Helene Skjøt-Arkil

**Affiliations:** 1 Research Unit in Emergency Medicine, Hospital of Southern Jutland, University of Southern Denmark, Aabenraa, Denmark; 2 Private Practitioner, Vojens, Denmark; 3 Emergency Department, Hospital of Southern Jutland, Aabenraa, Denmark; 4 Medical Department, Hospital of Southern Jutland, Aabenraa, Denmark; 5 Private Practitioner, Tonder, Denmark; 6 Private Practitioner, Soenderborg, Denmark

**Keywords:** Hospital-at-home, Community, Hospital, General practitioner, Hospital specialist, Elderly

## Abstract

**Background:**

Hospital at home (HaH) is an alternative to acute admission for elderly patients. It is unclear if should be cared for a primarily by a hospital intern specialist or by the patient’s own general practitioner (GP).

The study assessed whether a GP based model was more effective than a hospital specialist based model at reducing number of hospital admissions without affecting the patient’s recovery or number of deaths.

**Methods:**

Pragmatic, randomised, open-labelled multicentre parallel group trial with two arms in four municipalities, four emergency departments and 150 GPs in Southern Denmark, including + 65 years old patients with an acute medical condition that required acute hospital in-patient care. The patients were randomly assigned to hospital specialist based model or GP model of HaH care. Five physical and cognitive performance tests were performed at inclusion and after 7 days. Primary outcome was number of hospital admissions within 7 days. Secondary outcomes were number of admissions within 14, 21 and 30 days, deaths within 30 and 90 days and changes in performance tests.

**Results:**

Sixty seven patients were enrolled in the GP model and 64 in the hospital specialist model. 45% in the hospital specialist arm versus 24% in the GP arm were admitted within 7 days (effect size 2.7, 95% CI 1.3–5.8; *p* = 0.01) and this remained significant within 30 days. No differences were found in death or changes in performance tests from day 0–7 days between the two groups.

**Conclusions:**

The GP based HaH model was more effective than the hospital specialist model in avoiding hospital admissions within 7 days among elderly patients with an acute medical condition with no differences in mental or physical recovery rates or deaths between the two models.

**Registration:**

No. NCT02422849 Registered 27 March 2015. Retrospectively registered

## Background

The ageing of the global population is increasing the demand for medical services [1]. Although acute admission is the standard solution for treating acute serious illness, elderly patients have been recognised as being highly susceptible to functional decline, iatrogenic events, delirium, and hospital-acquired infections while hospitalised [2, 3]. Furthermore, adverse events occur during the hospital-to-home transition due to poor communication between the different sectors in the health care system [4].

There are substitutes to acute admissions, often referred to as hospital-at-home (HaH) service, defined as “a service that provides active treatment, by health care professionals, in the patient’s home of a condition that otherwise would require acute hospital in-patient care, always for a limited time period” [5]. In Denmark and other places the patient is sometimes offered a transfer from their home to the local nursing home for a short time period [6, 7].

These solutions have been evaluated in a range of randomised clinical trials [7, 8]. The included patients tend to have frequently occurring, relatively uncomplicated conditions, with well-defined treatments that could be delivered safely at home [7, 9]. Nursing care is always provided and physician inputs are available [7], either from a general practitioner (GP) [10, 11] or a hospital- specialist [6, 12]. The Patients might be admitted to HaH services either by the hospital specialist or the GP [5].

These solutions have been evaluated in a range of randomised clinical trials (RCT) comparing HaH with hospital admission [13]. Most of the studies favour the alternatives to hospital admission and demonstrate a reduction in subsequent admissions, faster recovery, and economic advantages [11, 14–17].

However, no studies have evaluated whether the patients in a HaH model should be cared for primarily by a hospital intern specialist or by the patient’s own GP. In a model, based on the hospital specialist, the patient will be initially transported to a hospital, examined by physicians, who are specialists in the conditions from which the patient is suffering with quick and easy access to advanced diagnostic procedures such as laboratory tests and diagnostic imaging, and the physician can easily confer with a variety of other specialists [18]. The hospital specialist will be responsible for the treatment and either visit the patient or otherwise be in contact with the patient and local community nurses during the next days. The hospital specialist may not be aware of the patient’s psychosocial conditions or familiar with the local community resources and health staff. In contrast, in a GP based model, the patient’s own GP might have the advantage of familiarity with the patient’s life situation and can follow the patient closely during the acute course of treatment, but may have less access to advanced diagnostic facilities or knowledge and experience at the specialist level.

Acknowledging these advantages and disadvantages, it is not obvious if the patient’s own GP or the hospital specialist should be responsible for the patients in a HaH setting. No randomised clinical trials have investigated this aspect so far. We thus did a pragmatic, randomised controlled trial (Acute Combined CarE for Seniors in Southern Jutland, (ACCESS)) to evaluate whether the patient’s own GP is more effective than a hospital specialist at reducing hospital admissions without affecting the recovery or death rates in elderly patients with acute medical conditions cared for in a HaH setting where the local community provides the nursing resources.

## Methods

### Study design and participants

In order to reflect clinical practice and to ensure generalisability we conducted a pragmatic, randomised, multicentre parallel group trial with two arms between 1 November 2013 and 30 June 2015 [19]. Four municipalities and four hospital emergency departments were involved, one in each municipality, covering a total of 150 GPs and 228,000 citizens in Southern Jutland, Denmark. All four municipalities had established HaH services, either in the patient’s home (Sønderborg and Haderslev) or in in the local nursing home (Tønder and Aabenraa).

The study was run through the research unit for Emergency Medicine at the University of Southern Denmark, Institute for Regional Research, Southern Centre, and was overseen by a trial steering committee. It was designed in accordance with the SPIRIT (Standard Protocol Items: Recommendations for Interventional Trials) 2013 Statement [20].

Patients with an acute medical condition that otherwise would require acute hospital in-patient care, were invited to participate in the study. They were identified by their GP or the municipal nurses who usually provided care for the patients.

We included patients aged 65 and over or 60 and over with significant comorbidity residing in one of the four municipalities. The patient’s own GP should be available the first two working days after inclusion. We excluded patients if they were assessed by someone other than their usual GP. Other exclusion criteria were if the patient was permanently residing in a nursing home, unable to give written consent, or had no relatives to give acting consent or if the capacity of the municipal care was fully utilised.

### Recruitment

Referral to the study was made through the GPs in their weekday opening hours. The GP was consulted either by the patient or the municipal nurse on the patient’s behalf concerning an acute medical problem. The GP then assessed the situation. If the GP was unable to see the patient immediately, a municipal nurse could be asked to visit the patient within an hour, regardless of whether or not the patient was already receiving municipal care services. The nurse would then assess the patient and report back to the GP. If the GP found, that the patient was in a condition that would require acute hospital in-patient care the patient was eligible for inclusion in the study. All 150 GP’s were allowed to refer patients to the project.

The GP or nurse informed the patient about the study both orally and in writing through prepared information documents. If the patient (or a relative in case of a temporarily or permanently mentally incompetent patient) agreed to be included, the GP or nurse called the randomisation centre and secured written consent.

### Randomisation and masking

The randomisation process was performed by an independent coordinating centre. The investigators, patients, GPs, and nurses could not influence to which group the patients were allocated. Participants were randomly assigned (1:1) to a hospital specialist HaH model or GP based HaH model with permuted blocks of 10, stratified in two groups, according to the municipal HaH model in the patient’s home (Sønderborg and Haderslev) or in local nursing homes (Tønder and Aabenraa). Allocations were prepared by an independent statistician and placed in serially numbered, opaque, sealed, tamper-evident envelopes by the independent coordinating centre. Treatment was determined by selecting the next randomisation envelope in sequence and was checked against a randomisation log. The correct and sequential use of envelopes as described in the protocol was strictly audited by the site research team and the independent coordinating centre. The result of the randomisation was communicated to the municipal HaH team, the GP or hospital specialist, and the patient. Treating clinicians could not be masked to the allocation. All analyses were done by investigators masked to treatment allocation.

### Procedures

For both groups, the HaH nursing care was performed by the municipally employed nurses. Before the recruitment of the patients, all municipal nurses completed a 1-day training programme and examination in assessment of the acute patient, including the use of the ADAPT triage system [21], in treatment and handling of the most frequently encountered acute conditions and in communication to other health professionals. An optional training programme in basic HaH service was offered to the all GP’s during two evening sessions, in which 94 GPs participated. Since the hospital doctors had not been involved in any former HaH programmes, eight physicians, all specialists in internal medicine, participated in the study and they all received similar training like the GPs.

All of the included patients were offered the same nursing care, regardless of whether or not the care took place in the patient’s home or at a nearby nursing home. In order to ensure equal treatment and care in the municipal HaH services, “minimum professional standard requirements” was defined, which included visits by the nurses up to eight times daily, administration of IV fluid and IV medicine, inhalation therapy, measurement of vital values, and triage.

The patients, who were randomised to the hospital specialist arm, were transported to the nearest hospital department and assessed by one of the eight participating specialists, who prescribed blood tests and imaging studies within 30 min after arrival. A detailed plan for the treatment and observation was decided within 4 h based on the results from the clinical examination and investigations. The plan was immediately sent electronically to the municipal HaH nurses. The hospital ensured that the treatment could be implemented immediately by transporting the patient to the HaH destination together with medical supplies, such as medicine and IV fluids, as prescribed and agreed with the HaH nurses. The specialist was responsible for the treatment, and one of the hospital specialists could be contacted directly by telephone within the next 48 h but was unable to visit the patient at home or at the nursing home. The GP was to be contacted for other medical issues not related to the HaH service.

The patients randomised to the GP arm were either seen in their own GP’s consultation or at home, depending on the situation. The GP examined the patient and performed a limited number of laboratory tests, such as C-reactive protein, B-glucose, hemoglobin concentration, and urine stix for urinary tract infection. Based on these findings, the GP prescribed a treatment and observation plan, which was immediately electronically available to the HaH nurses. The GP was responsible for the treatment prescribed and could be contacted by telephone during daytime. Outside working hours, the local GP on duty could be consulted. If the GP prescribed IV treatment, municipal services brought medicine and IV fluids to the patient.

The HaH service lasted up to 48 h after the inclusion. By this time, the municipal nurses and the physician caring for the patient had to decide whether the patient could return to usual GP care, including normal or increased municipal services, or should be admitted to hospital.

The participants were followed for 3 months after the inclusion time and subsequent hospital admissions and deaths were recorded.

The patients were tested with a range of performance tests at the time of inclusion and after 7 days: The 30-s chair-stand test as an indicator of lower body muscle strength and functional capacity (number of stands from chair in 30 s, range 0- stands/30 s) [22], the Morton Mobility Index (DEMMI) to measure mobility (15 items measures mobility and balance across the spectrum.

from bed-bound to independent mobility, score range 0–100) [23, 24], Orientation-Memory-Concentration (OMC) to evaluate cognitive performance (6 tests of memory, orientation and concentration, range 0–28) [25], and hand grip strength to provide a measure of the patient’s total strength (Jamar hand dynamometer, range 0–90 kg) [26, 27]. QALYs (quality-adjusted life years) were estimated using the self-completed EQ-5D instrument [28] and Danish preference weights (5 questions concerning mobility, self care, activity, pain and depression, weighted range 0–1). All of the tests were performed at the same time in the order: EQ-5D, DEMMI, OMC, chair-stand test, and grip strength. The completion of the tests was supervised by project assistants (nurses or physiotherapists) specifically trained for the assignment and not involved in the care of the patients. It was secured that the same assistant did not test the patient twice and there was no access to former test results. A high inter-rater reliability was secured by an experienced project physiotherapist, who instructed and trained all project assistants in the performance of the tests during a one-day course and controlled each assistant by supervision of their first performance and again during a control visit after 5 months. For the different tests (DEMMI, chair-stand and hand grip-test) good-excellent agreement was found.

### Outcomes

The primary outcome was the number of patients with a hospital admission within 7 days after the inclusion. Secondary outcomes were number of patients with a hospital admission within 14,21, 30 and 90 days after the inclusion, median admission days among the patients admitted within 7 and 90 days after the inclusion, number of patients who died within 30 and 90 days after the inclusion. We also measured the mean changes in the mental and physical performance tests and patient perceived quality of life score from day 0 to day 7. Data concerning hospital admissions and death were obtained from the Danish National Patient Register and by reviews of the patient files.

### Statistical analysis

All data collected for this study were entered directly into electronic predesigned forms by using computer tablets and stored in accordance with the Danish Data Protection Agency requirements.

The RCT was stratified into two layers, depending on the municipal HaH care. The analyses were performed at an accumulated level.

To calculate the required sample size, we estimated a 40% admission rate in the community arm on the basis of local audit data. We powered our study to detect at least a 25% absolute reduction in admissions in the hospital specialist arm compared to the GP arm, using the minimum improvement we thought would effect a change in practice. We estimated that this difference would require 82 patients per group, 164 patients total, which would allow the analysis of the study at an aggregated level assuming a two-tailed test of statistical significance with an α of 0.05 and power of 0.8.

The data was analysed according to intention-to-treat principles. We report baseline data descriptively by group and for simple comparison of categorical variables we used Fisher exact test and for continuous variables Kruskall-Wallis test. For the analysis of the outcomes we compared binary outcomes (including the primary outcome) using logistic regression and continuous outcome using linear regression with allocation group as a fixed effect and municipals as a random effect. All patients had complete data for the primary outcome and death. For the secondary outcomes multiple imputation method was using the variables age, gender, municipal, residence, home care and triage to replace the missing data for the following outcomes: DEMMI score, Grip Strength test, OMC and RSS test (33% missing) and EQD5 (44% missing), The statistical analyses were performed in STATA v. 14. The reporting of the results is in accordance with CONSORT 2010 statement.

## Results

**Fig. 1:**
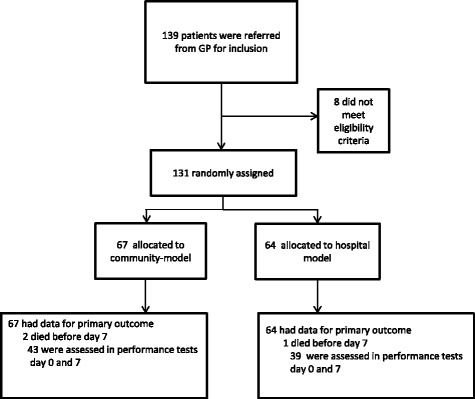
Flow diagram for the ACCESS study

Between November 2013 and February 2015, 139 patients were referred from 72 (43% of all) GPs for the study and 131 patients were found eligible and included. We randomly assigned 67 patients to the community arm and 64 patients to the hospital arm (Fig. 1). Eight hospital specialists and 44 GPs delivered the intervention.*.*

No patients were lost to follow-up. Baseline demographic, reasons for referral, triage, performance tests, and perceived quality of life were similar in each group. The patients in both arms were suffering from a range of acute conditions commonly found in this age (Table 1).

**Table 1:** Baseline Characteristics

	Community model (*n* = 67)	Hospital model (*n* = 64)	*P* value
Men	23 (34%)	24 (38%)	0.72
Age (years)	83 (77–88)	84 (77–89)	0.48
Residence			0.76
Own home alone	38 (61%)	32 (63%)	
Own home with spouse	22 (36%)	16 (31%)	
Sheltered housing	2 (3%)	3 (6%)	
Home care			0.22
No home care	15 (22%)	11 (17%)	
Home care	44 (66%)	38 (59%)	
No information	8 (12%)	15 (23%)	
Reason for referral (1)			
Acute exacerbation of COPD	11 (17%)	7 (11%)	0.45
Dehydration	11 (17%)	18 (28%)	0.14
Delirium	5 (8%)	4 (6%)	1.0
Fever	6 (9%)	3 (5%)	1.0
Pneumonia	20 (30%)	13 (20%)	0.23
UTI	6 (9%)	10 (16%)	0.30
Other	29 (44%)	27 (42%)	0.86
Triage			0.99
Green (not urgent)	34 (54%)	28 (55%)	
Yellow (standard)	16 (25%)	14 (27%)	
Orange (urgent)	10 (16%)	7 (14%)	
Red (very urgent)	3 (5%)	2 (4%)	
Function tests			
DEMMI (*n* = 50 /41)	40 (27–53)	39 (24–57)	0.77
OMC (*n* = 50/41)	20 (11–24)	20 (14–24)	0.71
CTS (*n* = 50/41)	0 (0–5)	0 (0–5)	0.76
GST (*n* = 50/41)	13 (6–24)	16 (4–24)	0.58
Quality of life			
EQ5D (*n* = 55/45)	0.59 (0.45–0.65	0.54 (0.36–0.66)	0.32

Data are n (%) or median (IQR). *COPD* chronic obstructive pulmonary disease, *UTI* urinary tract infection, *DEMMI* de Morton Mobility Index, *OMC* orientation-memory-concentration test. *CST* 30-s. chair stand test, *GST* Grip strength test (1) more than one reason possible

The included patients were old and fragile, with a median age of 84 years, 60% lived alone and received municipal homecare. During the follow-up period of 3 months 53% of the patients would experience a hospital admission and 33% of them die (Tables 1 and 2).

**Table 2:** Primary and secondary outcomes

Admission and death	Community model (*n* = 67)	Hospital model (*n* = 64)	Effect size OR (95% CI)	*P* value
Admission on inclusion day	10 (15%)	25 (39%)	3.7 (1.6–8.7)	0.003
Admission within 7 days	16(24%)	29 (45%)	2.7 (1.3–5.8)	0.01
Admission within 14 days	17 (25%)	30 (46%)	2.7 (1.3–5.7)	0.01
Admission within 21 days	19 (28%)	30 (48%)	2.4 (1.2–5.1)	0.02
Admission within 30 days	23 (31%)	33 (52%)	2.1 (1.0–4.3)	0.04
Admission within 90 days	33 (49%)	37 (58%)	1.4 (0.7–2.9)	0.30
Median admission day for patients admitted within 7 days (IQR)	1 (1–3)	0 (0–0)		0.001
Median days in hospital for patients admitted within 7 days	4 (3–8)	6 (1–10)		0.06
Median number of admissions within 90 days for admitted patients (IQR)	1 (1–2)	1 (1–2)		0.67
Median days in hospital with 90 days for admitted patients (IQR)	7 (4–11)	7 (3–13)		0.83
Death within 30 days	5 (7%)	7 (11%)	1.6 (0.4–5.9)	0.48
Death within 3 months	11 (16%)	12 (19%)	1.2 (0.5–3.1)	0.69
Change in performance (day 7)				
Increased municipal care	20 (39%)	30 (57%)	1.9 (0.9–4.3)	0.11
			Coeficent (95% CI)	
Mean DEMMI change (95% CI))	10 (2.6–17)	5 (−0.1–10)	−0.5(−1.4–0.5)	0.3
Mean OMC change (95% CI))	2.8 (− 0.1–5.7)	1.8 (− 0.8–4.4)	−0.1 (− 0.9–0.7)	0.9
Mean CST change (95% CI)	0.4 (− 0.8–1.7)	0.2 (− 0.9–1.2)	−0.3 (− 1.3–0.6)	0.5
Mean GST change (95% CI)	0.1 (−3.4–3.5)	3.0 (− 0.5–6.5)	−0.3 (− 1.0–0.5)	0.4
Mean EQD5 change (95% CI)	0.1 (0.0–0.1)	0.1 (0.0–0.1)	−0.1 (− 0.8–0.6)	0.7

*DEMMI* de Morton Mobility Index, *OMC* orientation-memory concentration test, *CST* 30-s. chair stand test *GST* Grip strength test

The primary outcome was that almost twice as many patients were admitted in the hospital specialist arm, 29 (45%) versus 16 (24%) of the patients in the GP arm within the first 7 days (Effect size 2.7, 95% CI 1.3–5.8; *p* = 0.01). The absolute risk difference was 21%; thus, nearly five patients needed the GP based service to avoid one case of admissions within the first 7 days. This significant difference was also found at 14 days and 30 days, but no difference was seen after 90 days (Table 2). The hospital specialist decided to admit 39% of all the patients within the first day after inclusion, while the GP only admitted 15% within the same period (p: 0.003).

No significant differences between the groups were found in death within the next 3 months or in municipal care level, changes in function tests, or perceived quality of life 1 week after the inclusion (Table 2).

## Discussion

We have shown that among a group of old and fragile patients with acute medical complaints partipating in a HaH service a hospital specialist admitted almost twice as many patients to hospital as a GP (45% versus 24%) within the first 7 days after the onset of the disease. This difference was also seen 30 days after the onset of the acute condition. We did not detect any differences in 7 days recovery rate or perceived quality of life between the groups or in numbers of death within 30 or 90 days.

The strength of our study is that it was carried out as a pragmatic study in the setting where the patients are usually identified and handled, involving the community nurses, GPs, and the hospital. There are however, some important limitations. A major limitation is that we did not manage to reach the expected number of inclusions. While our main result, difference in numbers of admission on day 7, remained significant despite a lower number of patients than expected, we were unable to find significant differences after day 30. We included a range of potentially adverse outcomes, like differences in death rates and recovery rates and were not able to show any significant differences in these outcomes, which might be due to the limited number of included patients.

Another limitation is that it was not possible to conceal the randomisation allocation for the patients or the health care professionals. Furthermore, since 44 GPs were involved, the individual GP gained only little experience in the HaH concept, and further experience with HaH would likely result in even higher differences between the GP and hospital specialist model. Finally, not all patients were interested or mentally able to participate in the physical and mental function tests on day 0 and 7, which weaken these results.

This study is the first to evaluate a GP versus hospital specialist based HaH model, and we are unable to compare our results directly with other findings in a similar design. Of the many HaH publications, few have examined the same group of elderly with a broad spectrum of acute diseases as we studied [8]: Two British studies revealed admission rates of 21% and 35% in a GP-based HaH in the patient’s own home [11, 29], quite similar to our findings. Two other studies of hospital specialist based HaH services from New Zealand and Australia found that 21% and 19%, respectively, were admitted to hospital [8, 12, 30], which was lower than our findings. These four studies suggest that admission rates are lower in a hospital setting than in the GP setting, which was also our expectation. However, the hospital-specialist HaH studies were based on a comprehensive hospital-outreach service in the patient’s home, including follow-up visits from hospital doctors, which was not an option in our case.

Our results raise some questions which deserve to be discussed in more details. Firstly, were the GP based HaH care truly preventing or rather delaying admissions of these patients? The prevention of hospitalisation seems to last for some weeks only. The design of the study does not allow us to judge whether later admissions were related to the acute illness which caused the inclusion the study or were related to a new situation. On the other hand, no later excessive admissions were seen in the GP group within the next months. Since the patients were not allowed to be included more than once within the observation period, we do not know if repeated HaH solutions managed by the GPs would have avoided further admissions. It might be argued that in this late stage of life even a single avoided or delayed admission would be appreciated by the patient if alternatives to admissions are possible.

Secondly, was the high admission rate of patients in the hospital specialist arm merely a result of differences between a hospital specialist and a GP strategy? If the patient had already reached the outpatient department of the hospital, it might be safer for a specialist to resort to a “keep and watch” strategy, where the patient was admitted to hospital rather than returned to home. The high number of admissions on the inclusion day and short-stay admissions in the hospital arm may indicate this. Although the hospital specialists had been thoroughly introduced to the HaH alternative to admission most of them had little or no practical experience with management of patients in a HaH setting. Increasing experience with HaH concepts might in future reduce the hospital specialist’s preference for admission. In contrast, the patient’s own GPs had activated the HaH team and initiated a “carefully watch at home” strategy, where the patient was closely observed at home andthe majority of the admissions in the GP arm occurred one to 3 days after the inclusion. However, since the GP’s practical experience with HaH was also very limited, and only increased a little in this project due to the low number of patients per GP, additional experience in future might reduce the admission numbers even more in the GP based HaH models.

Our study has a number of clinical implications. In contrast to previous results and our expectations, the GP based model was more successful than the hospital specialist model in avoiding hospital admissions when caring for the elderly patients in the HaH setting. We believe that the GPs’ advantage of familiarity with the patient and close collaboration with the municipal care system outweigh the hospital specialists’ specific knowledge of the disease and access to advanced diagnostic procedures.

For the fragile, old patients with high morbidity and mortality, it is an exhausting experience to be transported to the hospital outpatient ED, assessed by specialists and passing through diagnostic procedures including blood tests and often diagnostic imaging procedures. As our results indicate this does not increase the recovery rate or reduce death rate compared to the simpler GP controlled HaH alternative, but merely increases the number of hospital admissions. As long as the community care is able to provide immediate HaH services this patient group is better handled by the community and own GP. However, before the community and GPs assume responsibility for some of the elderly patients in HaH models, many problems must first be solved. HaH models are complex interventions and require close collaboration between the GPs, the municipal care system, and the hospitals and involves resource allocation, economy, cultural and legal issues [31]. In Denmark, for instance, resources and funds need to be transferred from the secondary health care, the hospitals, to the primary health care sector and GPs, if HaH models replaces hospital admissions. Furthermore, the patients and relatives need to be reassured that the HaH model is not an inferior health care solution, compared to a hospital admission. It also requires that the community nurses and GPs are properly trained in HaH care, and that legal aspects concerning the responsibility for the patients in HaH care is clear.

We believe that our results are also valuable outside Denmark in countries with similar health care systems. Future work should assess whether our findings are replicable in other community and own GP-based HaH models together with the economic consequences for the total health care system.

## Conclusion

In conclusion, we found that a GP based HaH model including the patient’s own GP was superior to a hospital specialist based HaH model in avoiding hospital admissions among elderly patients with acute medical diseases. There were no measurable differences in terms of mental or physical recovery rates or deaths between the two models.
